# Volumetric computed tomography analysis for gastroduodenal and pancreaticoduodenal artery aneurysm formation: A retrospective single-center study

**DOI:** 10.1097/MD.0000000000029539

**Published:** 2022-06-17

**Authors:** Ryohei Maeno, Katsuyuki Hoshina, Kazuhiro Miyahara, Masamitsu Suhara, Mitsuru Matsukura, Toshihiko Isaji, Toshio Takayama

**Affiliations:** Division of Vascular Surgery, Department of Surgery, Graduate School of Medicine, The University of Tokyo, Tokyo, Japan.

**Keywords:** celiac artery lesions, gastroduodenal artery aneurysms, hemodynamic change, pancreaticoduodenal artery aneurysms

## Abstract

Gastroduodenal artery aneurysms (GDAA) and pancreaticoduodenal artery aneurysms (PDAA) are rare, have high rupture risks, and are located in the arcade between the celiac artery and the superior mesenteric artery. Pancreaticoduodenal artery aneurysms are associated with celiac artery stenosis, and it is hypothesized that these celiac lesions might contribute to the formation of aneurysms. In contrast, a few studies have reported an association between a gastroduodenal artery aneurysm and celiac lesions. This study aimed to investigate the potential differences between patients with gastroduodenal and pancreaticoduodenal artery aneurysms and better understand their pathogenesis.

We selected patients with GDAA and PDAA who were admitted to our department between January 2010 and December 2020. Aortic wall volume, aortic wall calcification, and pancreaticoduodenal arcade volume of computed tomography images were calculated semi-manually using Horos 3.3.5.

Eight GDAAs and 11 PDAAs were analyzed. Celiac lesions were found in all PDAA patients, with none in GDAA cases. Volumetry demonstrated that aortic wall volume and calcification were more prominent in the GDAA group than in the PDAA group (*P* = .026 and *P* = .049, respectively). The pancreaticoduodenal arcade volume was larger in the PDAA group (*P* = .002).

In our study, celiac artery lesions were strongly correlated with PDAA. The volume of the pancreaticoduodenal arcade was larger in the PDAA group, and aortic wall volume and calcification were larger in the GDAA group.

## 1 Introduction

Gastroduodenal artery aneurysm (GDAA) and pancreaticoduodenal artery aneurysm (PDAA) have several common features: rarity, accounting for approximately 1.5% and 2% of visceral aneurysms, respectively^[1]^; high rupture risk, requiring intervention regardless of the aneurysm size^[2,3]^; and location in the pancreaticoduodenal arcade between the celiac artery and the superior mesenteric artery. PDAA is associated with celiac artery stenosis, and it is hypothesized that these celiac lesions contribute to PDAA formation. However, few reports have established a link between GDAA and celiac lesions.^[4]^

To investigate possible hemodynamic mechanisms involved in aneurysm formation, we previously created an electric circuit model of the pancreaticoduodenal arcade (PDA) and simulated the degree of change in blood flow with the degree of celiac stenosis.^[5]^ The PDA flow decreased to zero when the celiac artery (CA) stenosis increased from 0% to 50% and reversed and increased when the CA stenosis exceeded 50%, eventually increasing to 3 times the initial flow for stenosis of 90% or more. However, changes in the flow rate in the gastroduodenal artery (GDA) were less pronounced during similar modifications in the degree of CA stenosis.

Apart from hemodynamic differences, GDAA and PDAA may also differ in terms of mean patient age, with one case series suggesting that the mean age of GDAA patients was higher than that of PDAA patients.^[5]^ The unique features associated with GDAA (older age; less variable hemodynamics) might suggest that other factors, such as atherosclerosis, might contribute to GDAA formation. In contrast, PDAA may be more closely associated with hemodynamic factors.

This retrospective single-center study investigated potential differences between patients with GDAA and PDAA through volumetric analysis of computed tomography (CT) images. We analyzed arterial morphology to investigate the degree of underlying atherosclerosis and arterial remodeling due to CA stenosis.

## 2 Methods

### 2.1 Patients

We selected patients with GDAA and PDAA who were admitted to our department between January 2010 and December 2020, and 22 patients were eligible. Of these, 3 patients with genetic disorders were excluded.

All patients provided written informed consent prior to inclusion in the research, and the use of the images for the research was approved by the Ethics Committee of The University of Tokyo Hospital (No. 3316-[3], 3252-[5]).

### 2.2 Definition of arteries

**Figure 1:**
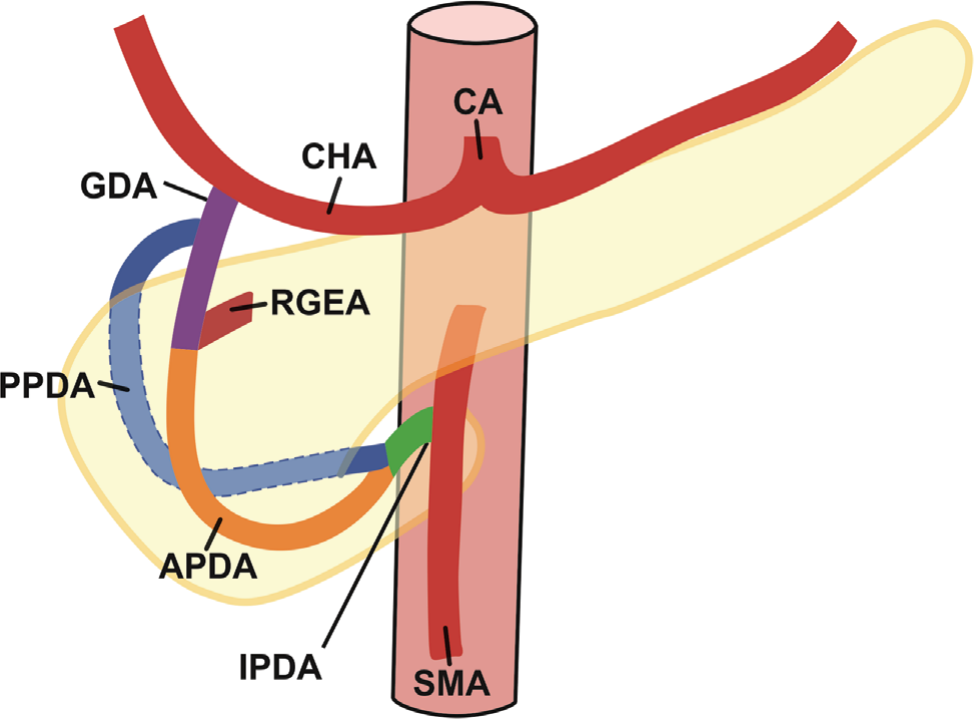
Anatomy of the pancreaticoduodenal arcade.

The gastroduodenal artery (GDA) extends from the bifurcation of the common hepatic artery to the right gastroepiploic artery, and we defined an aneurysm formed in this region as GDAA. PDA was defined as the collective term for anterior pancreaticoduodenal artery (APDA), posterior pancreaticoduodenal artery (PPDA), and inferior pancreaticoduodenal artery (IPDA). APDA was defined as the artery from right gastroepiploic artery to IPDA, PPDA was defined as the artery from GDA to IPDA, and IPDA was defined as the artery from the confluence of APDA and PPDA to the confluence of the superior mesenteric artery (SMA) (Fig. 1). PDAA was defined as an aneurysm in that region.

### 2.3 Variables

The following variables were recorded: patient age, gender, height, weight, body mass index (BMI), CA stenosis, SMA stenosis, aneurysm size, presence of rupture, treatments, follow-up period, and aneurysm recurrence. CA and SMA stenosis were defined as the presence of more than 50% stenosis on axial CT imaging using the North American Symptomatic Carotid Endarterectomy Trial method.

### 2.4 Volumetry

**Figure 2:**
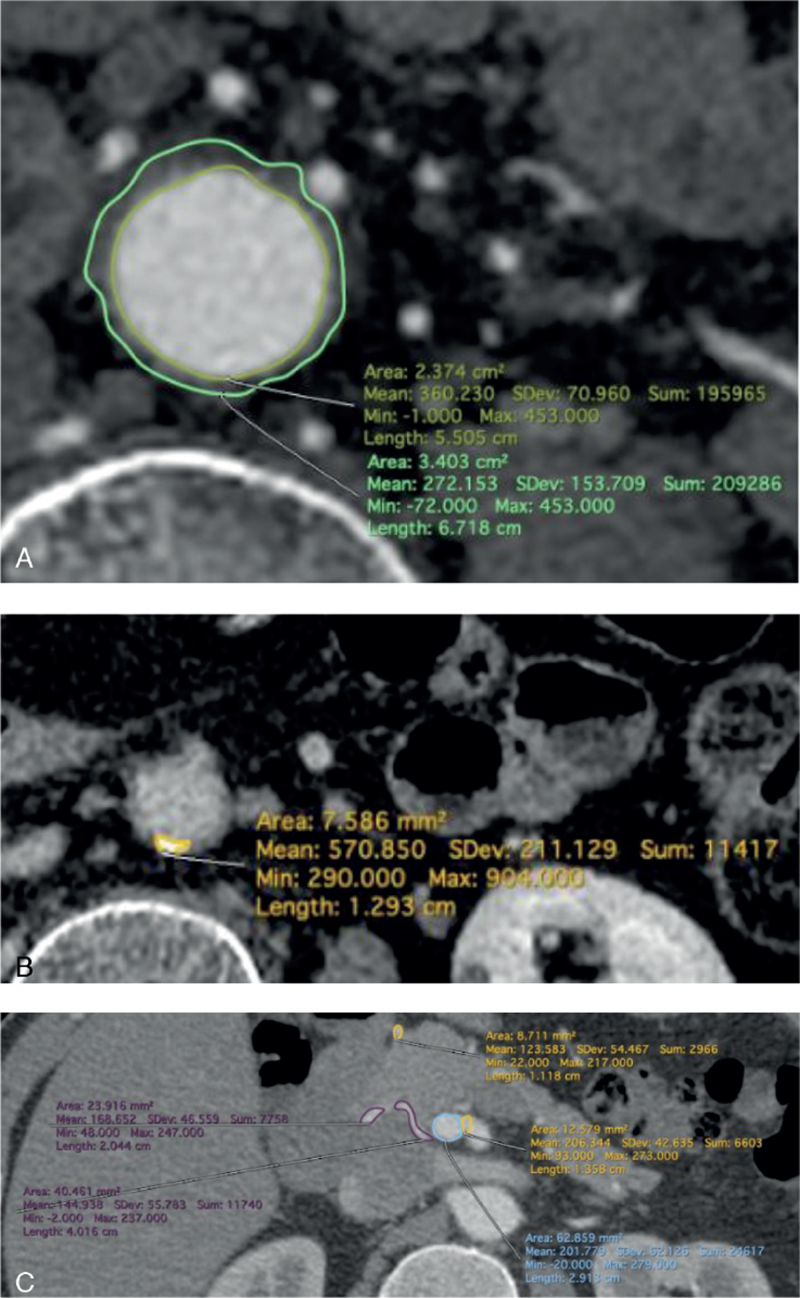
Volume calculation of CT images using Horos 3.3.5. (A) Volume of the aortic wall. (B) Volume of calcification. (C) Volume of pancreatic arcade vessels.

The volumes of the aortic wall, aortic wall calcification, and PDAs in thin-sliced (1 mm) CT images were calculated using Horos 3.3.5, an open-source medical image viewer. We identified the areas manually and summed the calculated volumes of all slices (Fig. 2). We measured and summed aortal volume from the celiac axis to the aortic bifurcation (Fig. 2A). Aortic wall calcification was defined as an area with more than 250 Hounsfield units (Fig. 2B). The volume of the pancreaticoduodenal arcade was calculated by adding the volumes of GDA and PDA and subtracting the aneurysm volumes (Fig. 2C). These were measured by 2 vascular surgeons, and the averaged values were calculated.

### 2.5 Statistics

Statistical analysis was performed using the statistical software EZR (version 1.54). The Mann–Whitney *U* test was used to compare age, height, weight, BMI, aneurysm size, aortic wall volume, aortic wall calcification volume, and pancreatic arcade volume. Values were reported as mean ± interquartile range (IQR). Gender and CA stenosis were compared using Fisher exact test. Inter-rater reliability was reported as intraclass correlation coefficient and 95% confidence interval (CI). Statistical significance was set at *P* < .05. Correlation between the GDAA diameter and the rupture were analyzed using Fisher's exact test and values were reported as mean ± standard deviation.

## 3 Results

There were eight cases of GDAA and 11 cases of PDAA, after excluding one case of Marfan syndrome and 2 cases of segmental arterial mediolysis in the PDAA group. The median age of patients with GDAA tended to be higher than those with PDAA (67.5 years, IQR: 54.8–71.3; 55.0 years, IQR: 47.0–70.5), without statistical significance (*P* = .363). There was no difference in gender, body size, including height, and weight, hypertension and smoking history between the 2 groups (Table 1). Celiac lesions were found in all PDAA patients (n = 11), with none found in GDAA cases. No SMA stenosis was found. The average aneurysm diameter was not statistically different between the GDAA and PDAA groups (9.0 mm, IQR: 6.3–12.8; 9.5 mm, IQR: 7.8–12.8). A rupture was found in 3 PDAA cases (27.3%). Treatments in the 2 groups were as follows: 5 coil embolizations and 3 surgical resections (GDAA); 5 coil embolizations and 2 surgical resections with 1 concomitant bypass (PDAA). There was no aneurysm recurrence during the follow-up periods for the GDAA and PDAA groups (21.0 months, IQR: 8.8–31.5; 35.0 months, IQR: 15.5–58.0).

**Table 1:** Patients’ demographics.

	GDAA (n = 8)	PDAA (n = 11)	*P* value
Age (years, IQR)	67.5 (54.8–71.3)	55.0 (47.0–70.5)	.363
Male gender n (%)	4 (50.0)	6 (55.0)	1.000
Height (cm, IQR)	161.3 (153.0–163.9)	163.0 (155.5–167.9)	.409
Body weight (kg, IQR)	62.3 (55.9–71.4)	61.0 (51.6–67.3)	.904
Hypertension (%)	3/11 (27.0)	6/8 (75.0)	.070
Smoking (%)	5/11 (45.4)	4/8 (50.0%)	1.000
BMI (kg/m^2^, IQR)	24.0 (20.9–26.8)	23.2 (21.1–26.3)	.600
Celiac artery stenosis n (%)	0 (0.0)	11 (100.0)	<.001
Aneurysm size (mm, IQR)	9.0 (6.3–12.8)	9.5 (7.8–12.8)	.509
Rupture n (%)	0 (0.0)	3 (27.3)	.228
Treatment n	coil embolization 5surgical resection 2no treatment 1	coil embolization 5surgical resection 3surgical resection and bypass 2no treatment 1	
Follow-up period (months, IQR)	21.0 (8.8–31.5)	35.0 (15.5–58.0)	
Recurrence n (%)	0 (0.0)	0 (0.0)	
Volumetry (cm^3^, IQR)			
Aortic wall volume	14.6 (13.6–17.5)	11.6 (8.9–11.6)	.026
Aortic wall calcification volume	0.11 (0.07–0.21)	0.00 (0.00–0.07)	.049
Pancreatic arcade volume	0.69 (0.59–1.37)	3.00 (1.89–5.28)	.002

BMI = body mass index, GDAA = gastroduodenal artery aneurysm, IQR = interquartile range, PDAA = pancreaticoduodenal artery aneurysm.

There was a significant difference in CA stenosis (*P* < .001), with greater aortic wall volume and aortic wall calcification in the GDAA group than in the PDAA group (*P* = .026; *P* = .049). The pancreaticoduodenal arcade volume was larger in the PDAA group (*P* = .002) (Table 1). The intraclass correlation coefficient for aortic wall volume, aortic wall calcification volume, and pancreatic arcade volume were 0.90 (CI: 0.77–0.96), 0.95 (CI: 0.87–0.98), and 0.99 (CI: 0.97–1.00), respectively.

The aneurysm diameters of 3 ruptured and 9 unruptured PDAAs were compared. The mean aneurysm diameters were 7.7 ± 0.5 mm and 12.9 ± 6.7 mm in the ruptured and unruptured groups, respectively. All ruptured aneurysms were <10 mm, and 6 unruptured aneurysms were >10 mm. There was no significant correlation between the event of aneurysm rupture and the aneurysm diameter, with 10-mm diameter as the cutoff value (*P* = .182).

## 4 Discussion

This study demonstrated a strong association between PDAA and celiac stenosis, with all PDAAs having concomitant celiac lesions. No such association was found in GDAA. Previous studies showed a correlation between blood flow in the PDA region and severity of celiac stenosis^[5]^ and demonstrated remodeling in the PDA region using the one-dimensional cardiovascular model.^[6]^ To investigate the clinical effect of the celiac lesion on arterial remodeling, we calculated the volume of the PDA and found it to be higher in the PDAA group. In contrast, the larger aortic wall volume and calcification in the GDAA group might support our hypothesis that atherosclerosis is a prominent factor in GDAA formation than the hemodynamic effects of celiac stenosis.

The association between CA stenosis and PDAA was first reported by Sutton and Lawton,^[7]^ who proposed the widely accepted hypothesis that PDAA develops due to increased collateral blood flow to the PDA caused by CA stenosis. However, it has been challenging to obtain evidence for their proposition due to the rarity of PDAA and the wide variation in peri-pancreatic communicating arteries.^[8]^ The usual vascular arcades comprise the anterior superior pancreaticoduodenal artery, posterior superior pancreaticoduodenal artery, anterior inferior pancreaticoduodenal artery, and posterior inferior pancreaticoduodenal artery. However, a “missing arcade” (absence of Anterior superior pancreaticoduodenal artery-Anterior inferior pancreaticoduodenal artery or Posterior superior pancreaticoduodenal artery-Posterior inferior pancreaticoduodenal artery anastomosis) has been reported in 5% to 50% of people.^[8]^ Therefore, Sutton and Lawton's hypothesis does not necessarily apply in actual clinical cases of GDAA and PDAA.

Although we propose that atherosclerosis contributes to GDAA formation, a case report of GDAA with SMA stenosis has illustrated the effects of hemodynamics on aneurysm formation.^[9]^ Theoretically, blood flow might increase to compensate for the decreased supply from the SMA. Although most celiac lesions are caused by median arcuate ligament syndrome (MALS), most SMA lesions are caused by SMA dissection. Furthermore, atherosclerosis is considered to be one of the causes of SMA dissection.

Time-lapse from disease onset can help elucidate the mechanism of aneurysm formation. In cases where MALS caused PDAA formation, it likely takes many years for hemodynamic factors to affect arcade arteries. Therefore, we do not think it is necessary to treat celiac lesions after the treatment of PDAA. Although there are reports of median arcuate ligament dissection and the prevention of aneurysm recurrence through celiac lesion revascularization,^[4,10]^ there was no recurrence in this study despite no intervention in MALS. Long-term follow-up is necessary if 1 adopts a conservative approach for intervention.

There are several limitations to our study. First, the sample size is small due to the rarity of these aneurysms. Our cohort of 8 cases of GDAA is too small to draw inferences about pathogenesis. Second, volumetry was performed manually, which may have led to the introduction of errors. Third, this study has limitations inherent in all retrospective studies, such as errors due to confounding and bias. Finally, the presence of systemic illnesses in this cohort may limit the generalizability of our findings.

## 5 Conclusion

In this retrospective single-center study analyzing 8 GDAAs and 11 PDAAs, CA lesions were strongly correlated with PDAA. The volume of the pancreaticoduodenal arcade was larger in the PDAA group, and aortic wall volume and calcification were larger in the GDAA group. These results might support the hypothesis that CA lesions have hemodynamic effects on the arcade through arterial remodeling and that atherosclerosis might play a more prominent role in GDAA formation than PDAA formation.

## Acknowledgments

I am grateful to Dr. Katsuyuki Hoshina, Kazuhiro Miyahara, Masamitsu Suhara, Mitsuru Matsukura, Toshihiko Isaji, and Toshio Takayama for helpful discussions and comments on the manuscript. And I would like to thank Editage (www.editage.com) for English language editing.

## Author contributions

**Conceptualization:** Katsuyuki Hoshina, Kazuhiro Miyahara, Ryohei Maeno.

**Formal analysis:** Ryohei Maeno.

**Investigation:** Ryohei Maeno.

**Methodology:** Kazuhiro Miyahara, Ryohei Maeno.

**Project administration:** Katsuyuki Hoshina.

**Supervision:** Katsuyuki Hoshina, Masamitsu Suhara, Mitsuru Matsukura, Toshihiko Isaji, Toshio Takayama.

**Validation:** Katsuyuki Hoshina, Ryohei Maeno.

**Visualization:** Ryohei Maeno.

**Writing – original draft:** Ryohei Maeno.

**Writing – review & editing:** Katsuyuki Hoshina.
